# *In vitro* model of bone to facilitate measurement of adhesion forces and super-resolution imaging of osteoclasts

**DOI:** 10.1038/srep22585

**Published:** 2016-03-03

**Authors:** Takahiro Deguchi, Maria H. Alanne, Elnaz Fazeli, Katja M. Fagerlund, Paula Pennanen, Petri Lehenkari, Pekka E. Hänninen, Juha Peltonen, Tuomas Näreoja

**Affiliations:** 1 Laboratory of Biophysics, Department of Cell Biology and Anatomy and Medicity Research Laboratories, University of Turku, Turku, Finland; 2 Department of Cell Biology and Anatomy, University of Turku, Turku, Finland; 3 Pharmatest Services Ltd, Turku, Finland; 4 Department of Anatomy and Cell Biology, Institute of Biomedicine, University of Oulu, Oulu, Finland; 5 Department of Neuroscience, Karolinska Institutet, Stockholm, Sweden

## Abstract

To elucidate processes in the osteoclastic bone resorption, visualise resorption and related actin reorganisation, a combination of imaging technologies and an applicable *in vitro* model is needed. Nanosized bone powder from matching species is deposited on any biocompatible surface in order to form a thin, translucent, smooth and elastic representation of injured bone. Osteoclasts cultured on the layer expressed matching morphology to ones cultured on sawed cortical bone slices. Resorption pits were easily identified by reflectance microscopy. The coating allowed actin structures on the bone interface to be visualised with super-resolution microscopy along with a detailed interlinked actin networks and actin branching in conjunction with V-ATPase, dynamin and Arp2/3 at actin patches. Furthermore, we measured the timescale of an adaptive osteoclast adhesion to bone by force spectroscopy experiments on live osteoclasts with bone-coated AFM cantilevers. Utilising the *in vitro* model and the advanced imaging technologies we localised immunofluorescence signals in respect to bone with high precision and detected resorption at its early stages. Put together, our data supports a cyclic model for resorption in human osteoclasts.

Bone remodelling is required for replacement of old bone with new; in order to sustain the biological function of the tissue, to repair damaged foci and to maintain Ca^2+^ homeostasis^1^,^2^. Imbalance in the remodelling leads to diseases like osteoporosis. Studies of osteoclast (OC) activities have been carried out on substrates like glass, plastic, hydroxyapatite, cortical bone slices, and dentin; and depending on culture substrate the OCs exhibit different morphology^3^,^4^. All cells adapt to and are influenced by the physical and chemical properties of their surroundings and constantly probe and extract information from the extracellular matrix (ECM)^5^,^6^. Several reports have examined the role of biological components^7^, chemical composition^8^, crystal structure, and grain size of biomaterials^9^ and their effect on OC bone resorption^10^,^11^,^12^,^13^. Many bone components have been shown to promote formation resorption-indicating structures in OC cultures^3^,^8^, but recent studies have identified circumstances questioning the relevance of these observations^14^,^15^. The experimental environment *in vitro* used to study a biological process should mimic the *in vivo* microenvironment to justify extrapolation of the results of an experiment back to the appropriate biological context^16^. In bone biology, it is often impossible to visualise protein localisation and processes in calcified matrix in molecular detail, especially in *in vivo* models^17^,^18^, and this prompts the development of more accessible natural-like *in vitro* models.

Bone resorption and polarisation of OC starts with the adhesion onto bone surface. The adhesion of OCs involves transmembrane molecules CD44 and α_V_β_3_ integrin, which interact directly and indirectly with extracellular matrix, and with intracellular, talin, vinculin and f-actin filaments^19^,^20^. These molecules are components of both podosomes (PD) and sealing zones (SZ)^21^,^22^,^23^. PDs are subcellular adhesion sites between OCs and ECM. The SZ refers to a functional subcellular structure that attaches the OCs to the bone surface and encircles the area being resorbed. SZ adhesion to bone has been characterised in a number of studies, but full mechanistic understanding of its function remains elusive^24^,^25^,^26^,^27^,^28^. The SZ has been claimed to develop from an actin patch (AP) on surface composed of apatite and collagen I^3^ and similar mechanism was suggested to take place on bone^26^. The ruffled border (RB) with folded membrane facing a resorption pit (RP) has been shown to the bone dissolving organelle^14^. Vacuolar proton pumps are shown to localise at the RB and it has been shown to undergo active vesicular traffic^29^,^30^,^31^. In many cases RBs are surrounded by a SZ, but it has been shown that RBs form and reduced level of resorption occurs in absence of fully functional SZs^14^ and that despite forming SZs some cells are unable to degrade the organic bone matrix and resorb bone^15^. Spatial organisation and dynamics of reorganisation of these structures have been studied in some detail^32^, these studies have revealed multiple relatively fast resorption bursts as compared to the classical more stationary sequence of events^3^. To develop a detailed understanding of the resorption process and forces folding the membrane and driving the vesicular transport an organotypic *in vitro* –model allowing high-resolution dynamic imaging needs to be established.

The current *in vitro* cell culture models either use artificial substrates; have rough surface that does not allow quantitation of resorption or thickness that does not allow use of super-resolution imaging. Our aim has been to empower research in bone biology with a cell culture model that would facilitate research on patient derived cells in a setting that would be three-dimensional, natural-like and accessible for modern imaging technologies. In the present study, we developed a method for bone powder coating of cell culture substrata and cantilevers of atomic force microscope (AFM). The experimental setup enables super-resolution imaging with stimulated emission depletion (STED) microscope, due to less dispersion from scattering, less autofluorescence, and less impact from the mismatch of refractive indices; and biophysical adhesion force measurements of OCs binding to bone. In OCs cultured on the coating we were able to examine processes at the onset of resorption, visualise actin micro-domains directly above RPs and establish a vesicular transport mechanism utilising polymerisation of branched actin^33^.

## Results

### Smooth, elastic and translucent *in vitro* model of injured bone

**Figure 1:**
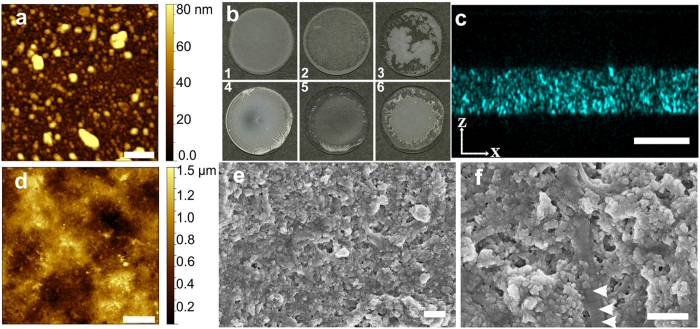
Thin, smooth and optically accessible 3D-culture model for bone biology research made from bone particles. AFM image of human bone particles (**a**) used in bone coating dried on a coverslip in low density. Appearances of bone particle coated glass coverslips (**b**) with 6 different coating buffer compositions: 1) 10 mM PO_4_, 0.05% BSA and 0.025% Triton X-100, 2) ethanol, 3) ion exchanged water, 4) 10 mM PO_4_ and 0.1% milk, 5) 10 mM PO_4_, 0.1% milk and 0.1% Triton X-100, and 6) 10 mM PO_4_ and 150 mM NaCl. A cross section image of bone particle coating acquired with laser scanning confocal microscope demonstrating the uniform thickness of the layer (**c**). Data was captured by setting a detector to capture reflected light, with excitation wavelength 476 nm. An AFM image captured in acoustic oscillation mode illustrates smoothness of the densely coated bone layer (**d**). Electron micrographs of bone coating at different magnifications, showing smooth surface (**e**) and type-I collagen fibril (**f**). The presence D-bands on the collagen fibrils (arrowheads in **f**) suggest that also the organic components of the bone are present and folded in native conformation. Scale bar 1 μm in (**a**), scale bar 10 μm in (**c**,**d**), 400 nm in (**e**,**f**).

**Figure 2:**
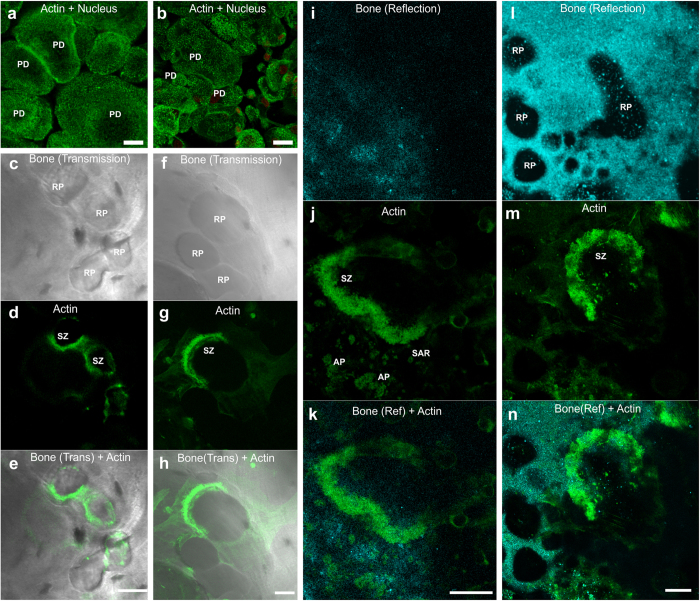
OC morphology on different substrata. Peripheral blood-derived OC morphology on (**a**) glass, (**b**) vitronectin coated glass, (**c–e**) sawed human bone slice and (**i–k**) human bone particle coating. On the glass-based substrates (**a**,**b**) the OCs are typically larger with more OCPs fused together and actin exhibits predominantly PD-structures, organised in rafts and rings. On the bone-derived substrates (**c**–**n**) OCs are induced to resorb bone that can be visualised from the presence of RPs. The average size of OCs is smaller and actin exhibits more complex patterns: wide actin belts represent SZs, actin patches (APs) with high but uneven phalloidin staining were aligned with RPs and small circular structures <5 μm in diameter (SAR). Bone marrow derived CD34+ OCPs on sawed human bone slice (**f**–**h**) and human bone particle coating (**l**–**n**) exhibit high resorption activity as demonstrated by a number of RP on the substrata. For both of the features PDs and SZs with both blood- and bone marrow derived OCs grown on the different substrates, the representative features were selected from N > 20 examples. Scale bar 20 μm.

To study bone-resorbing OCs in molecular detail, we produced a natural-like *in vitro* model of bone from milled nanoparticulate bone. The milling of cortical human bone and subsequent fractionation by sedimentation yielded a continuous size distribution of bone particles from 10–200 nm (Fig. 1a). The smoothest and most uniform coating over entire coverslip area was obtained with a coating buffer composed of 10 mM PO_4_, 0.025% (w/v) Triton X-100, and 0.05% (w/v) BSA (Fig. 1b1). Moreover, the BSA added to the buffer provided elasticity for the coating that enabled it to sustain bending of the AFM cantilever without breaking, while conducting force spectroscopy (FS) experiments. The bone-coated coverslips were imaged with LM, SEM and AFM to provide detailed characterisation of the created *in vitro* model of injured bone (Fig. 1). The x-z cross-sectional reflection image taken with confocal LM (Fig. 1c) shows a flat and smooth surface with an average thickness of the bone coating 7.5 ± 2 μm. In STED microscopy the coating facilitated micrograph resolution that was not possible on bone chip (Supplementary Fig. S1). The AFM image of the bone-coated coverslip (Fig. 1d) shows a flat surface with a peak-to-valley difference of 1.2 μm over an area of 50 × 50 μm. The SEM images of the bone-coated surface (Fig. 1e) also show a flat and smooth coating surface. The higher magnification (Fig. 1f) showed a fibril displaying typical electron dense bands of fibrillar collagen, indicative of the presence of proteins of the natural bone matrix and their successful reconstitution. On this coating the RPs were easily detected and we observed a wide range of RP depths (Fig. 2).

### Osteoclasts present different morphology when grown on bone instead of glass

**Figure 3:**
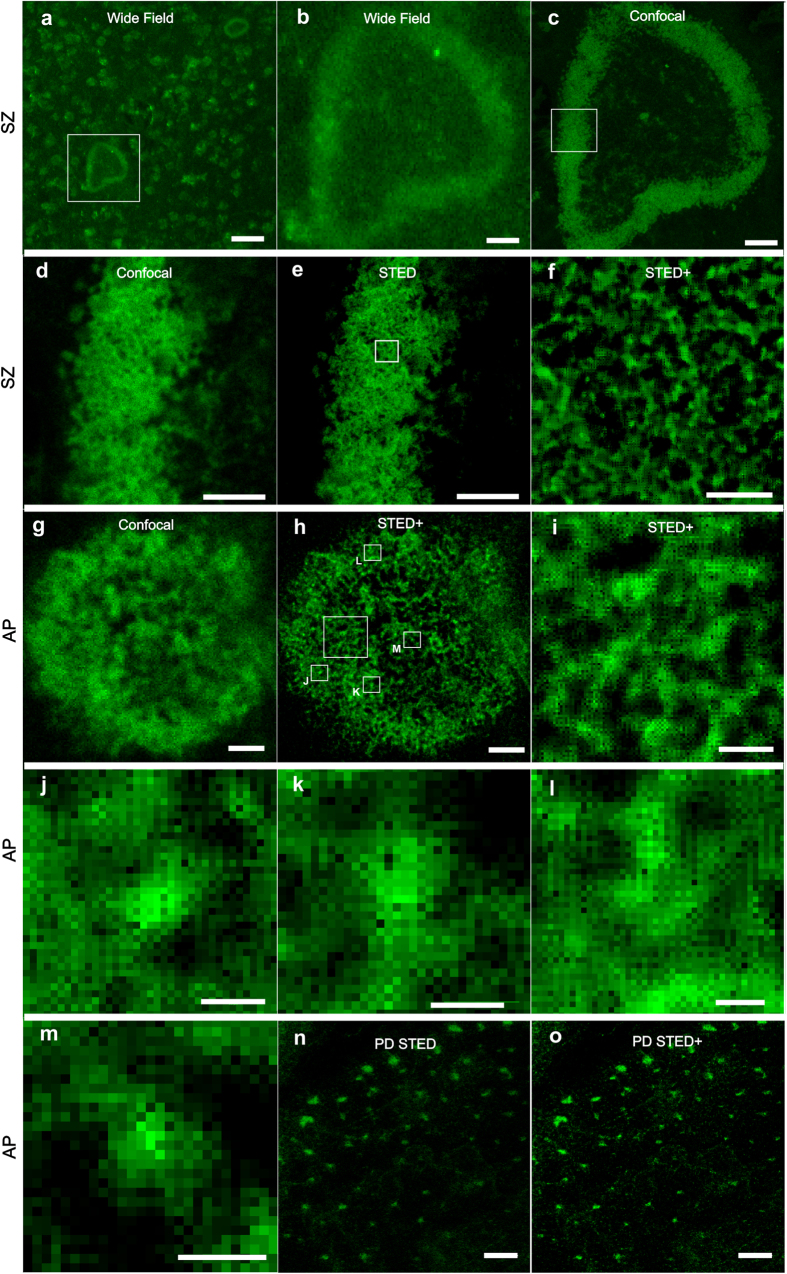
Super-resolution examination of phalloidin-stained actin structures in human OCs exhibiting SZs or APs. Panels (**a–f**) show SZ-structure at different magnifications captured with wide field (**a**,**b**), confocal (**c**,**d**) and STED (**e**,**f**) microscopes. STED+ shows deconvolved data. SZ exhibits a uniform dense actin staining with closely-knit structure of long parallel and crossing filaments. Whereas, AP (**g**–**m**) has a more network-like appearance with occasional brighter puncta. Here length of straight filaments is shorter and the network has more holes than in SZ. In high magnification of the deconvolved STED images from AP (**j–m**), on most of the bright puncta visualised we noticed a small hole that may correspond to an endocytosis event where a vesicle is budding from RB membrane by branched actin polymerisation. The representative SZ and AP was selected from N > 20 observed cases. Scale bar: 50 μm in (**a**), 10 μm in (**b**,**c**), 5 μm in (**d**,**e**), 1 μm in (**f**), 2 μm in (**g**,**h**), 0.5 μm in (**i**), 0.2 μm in (**j**–**m**) and 2 μm in (**n**–**o**).

To study bone resorption by human OCs, we differentiated and cultured the CD34+ osteoclast precursor cells (Lonza) and peripheral blood mononuclear cells on glass coverslips, on coverslips coated with vitronectin, on coverslips coated with human bone particles and on human cortical bone slices (Fig. 2). We were able to differentiate mature OCs on all substrates from both, peripheral blood derived monocyte fraction with RANKL – M-CSF treatment^34^,^35^ and from bone marrow derived OCPs by using the provider’s protocol. Confocal reflection and backscatter signals, recorded at the excitation wavelength, as well as images from transmitted light detector were used to visualise changes in the bone coating, i.e. RPs. Nuclear labelling and phalloidin labelling for f-actin were used to visualise the morphology of OCs and the presence of OC-specific actin structures, i.e. PDs, actin ring SZs and APs, a feature typically seen over a RP (Figs 2 and 3). The bone-derived substrates on which OCs were grown on had a distinct effect on the morphology of OCs (Fig. 2). On bone-derived materials the average size of OCs was smaller and there were more resorption related actin structures, whereas on glass there were larger round OCs with more PD-structures (Fig. 2). Actin features and overall morphology of OCs were similar on bone slices and bone coating (Fig. 2 and Supplementary Fig. S2). However, super-resolution imaging was not possible on sawed bone slice (Supplementary Fig. S1).

### Actin structures in OCs on bone derived materials

On vitronectin coated coverslips and uncoated coverslips (Fig. 2a,b) the in OCs were large, rounded and exhibited mainly PD-structures in the shape of both rings and rafts. In contrast, in OCs grown on bone there were fewer PDs, while other structures SZs, APs, and SARs were observed more frequently. Wide actin belts (width ≥ 2 μm, length and diameter 20–200 μm) were classified as SZs (Fig. 2d,g,j,m and Supplementary movies M1 and M2). The SZs could be found either at the edge of a RP or deeper inside of them organised in vertical orientation (Fig. 2d). When we examined the SZ in detail with STED microscopy, we observed a tightly packed lateral network of filaments (Fig. 3e,f). The organisation did not resemble the organisation of a PD-belt where star-shaped interlinked clusters were observed (Fig. 3n,o). Hence, formation of a SZ is likely to proceed through another mechanism than tighter clustering of PDs. Also we visualised actin networks termed APs of (diameter 5–50 μm) often directly above RPs (Fig. 2j, Supplementary Fig. S2B,E,H,K) and small (<5 μm in diameter) actin rings (SAR) (Fig. 2j, Supplementary Fig. S2H, K and video M2). Super-resolution imaging of the f-actin structure at AP revealed a network of interlinked filaments that were organised into convex pattern with some extending protrusions that closely align with the substrate (Fig. 3g–m). We visualised APs also at the bone interfaces where no clear RP or SZ was present (Fig. 2d, Supplementary Fig. S2C,E). This could suggest that the onset of resorption may not require a SZ to form prior to the formation of an AP-structure. Confocal images revealed an abundance of resorption pits on both bone slice and bone coating (Fig. 2c–l). The reflection light image from bone coating surface showed an otherwise smooth and uniform coating perforated with number of RPs (Fig. 2i,l, Supplementary Fig. S2 and Supplementary videos M1, M2 and M4), indicating active resorption. Some of these resorption sites consisted of a cluster of shallow small RPs, indicating dynamic resorption (Fig. 2). Consistent with previous EM studies^15^,^14^, we observed with DiI-staining that membranous structures are absent from the SZ and conversely we found overlap of membranous structures with f-actin at the AP (Supplementary Fig. S3 and M7).

### OCs fuse to larger cells on glass

**Figure 4:**
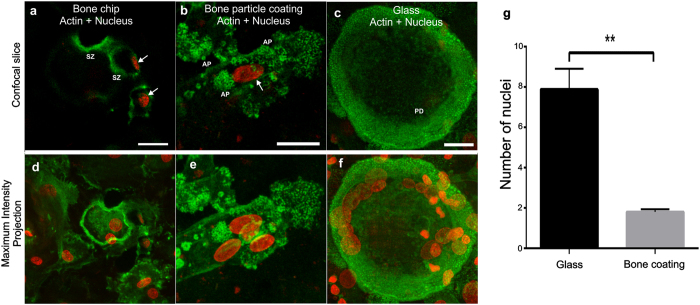
There are fewer nuclei in resorbing cells grown on bone coating than in multinuclear cells grown on glass. We counted the nuclei on three separate bone coated coverslips or clean coverslips, and found that on bone coating the OC precursors are less likely to fuse into large cells with many nuclei. In OCs associated with resorption pits, we typically found one nucleus tightly coupled within the actin structure, indicating a more active role in the transcription required in the resorption process. Resorbing peripheral blood-derived OCs on human bone slice (**a,d**) and human bone particle coating (**b**,**e**) with actin (green) and nucleus (red). Actin in (**a**) shows SZs with and in (**b**) AP both with a nucleus (arrows) tightly coupled with the resorptive zones. On glass (**c**,**f**) the nuclei are located high above the plane where PDs are seen. There is a significant difference in the number of nuclei in resorbing cells cultured on bone coating and on glass (**g**). For both cases N > 100, to be counted on glass the cell needed to have more than one nucleus and on bone coating it needed to express resorption specific features of be located on top of a RP. Scale bar 20 μm.

On both glass and vitronectin coated surfaces (Fig. 2a,b, Supplementary video M1), cells were rounded with multiple nuclei; on glass 1–20 nuclei per OC an average of 9.7 (N > 100) located in the middle of the cell (Fig. 4c,f). On bone-derived substrates, the number of nuclei was much lower; on bone coating only 1–4 nuclei per cell and on an average of 1.8 nuclei (N > 100), and many resorbing cells had only one nucleus (Fig. 4a,b,d,e,g). Furthermore, both on bone slices and bone coating, in the OCs associated with RPs, we typically found one nucleus tightly coupled within the actin structure close to the bone interface, indicating a more active role in the transcription required in the resorption process (Fig. 4 and Supplementary videos M1, M2 and M3).

### Morphology of resorbing osteoclasts

**Figure 5:**
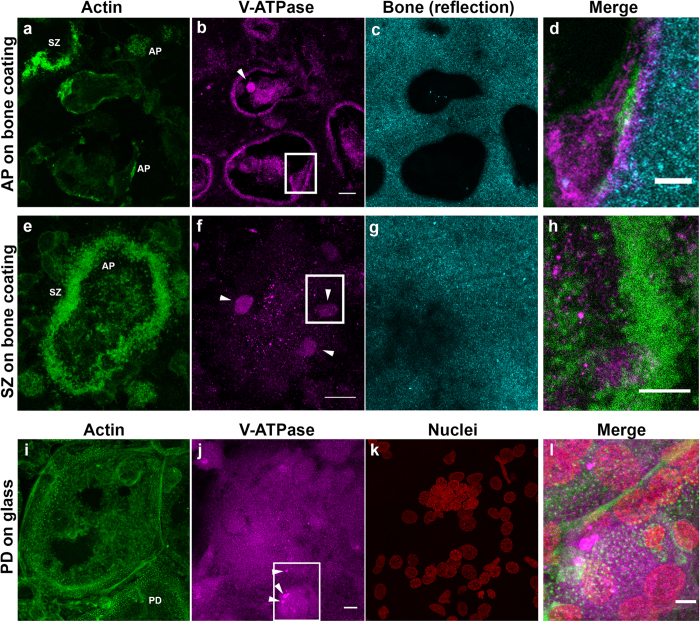
V-ATPase localisation in human OCs exhibiting APs, SZs and PDs. F-actin is displayed in green, V-ATPase in magenta and reflection from bone in cyan. (**a–d**) a 2.5 μm maximum projection of OCs above RPs with ongoing active resorption show an accumulation of V-ATPase at the bone interface. Actively resorbing cells are devoid of V-ATPase filled vacuoles, but have smaller puncta representing acidified endosomes and lysosomes. A 2.5 μm maximum projection (**e–h**) of an OC displaying a SZ structure, and a medium intensity of V-ATPase in the middle of the SZ. Further away from the bone interface are seen vacuoles filled with V-ATPase, along with endosomes and lysosomes. In a 5 μm maximum projection of OCs grown on glass (**i–k**) no accumulation V-ATPase was observed at the glass interface, in abundant endosomes and lysosomes and V-ATPase filled vacuoles were a typically seen stored inside the cell (bottom center). Boxes in (**b**,**f**,**j**) outline the field of view in the merge images (**d**,**h**,**k**). Arrowheads point to V-ATPase stained vacuoles. We selected the representative images from 18 V-ATPase stained SZs and N > 20 APs and PDs. Scale bar in (**a**,**e**,**i**)is 10 μm and in (**d**,**h**,**l**)5 μm.

**Figure 6:**
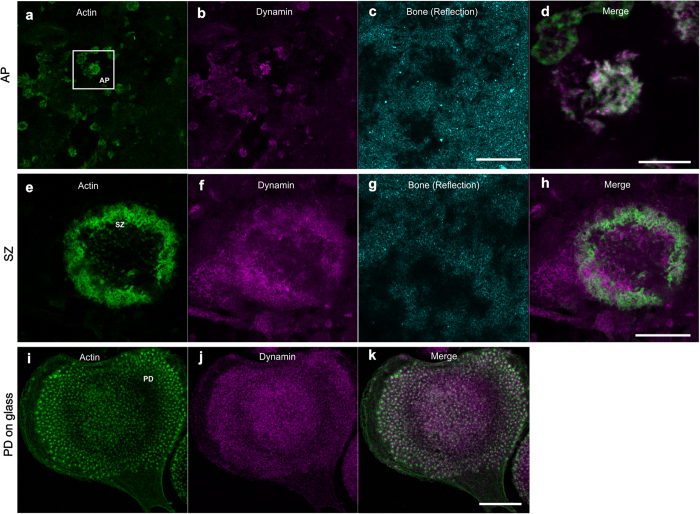
Dynamin localisation in human OCs exhibiting APs, SZs and PDs. (**a–d**) RP (bone surface in cyan) coincided with AP structures exhibiting a network of filamentous actin (green) and dynamin (magenta) staining. (**a**) actin at AP-structures was organised in form of intensely stained discs of networked actin with a number of bright puncta. A reflection signal image from the bone coating (cyan) where dark patches signify indentations i.e. RPs. (**b**) dynamin (mainly dynamin 2) showed staining throughout the plasma membrane and an intense staining at AP-structures, indicating specific accumulation at these zones and vesicular transport. (**d**) an overlay image of dynamin and actin shows dynamin localising at the puncta in actin network. An OC grown on bone coating exhibiting a SZ (**e–h**), does not show accumulation of dynamin at the SZ or at its center. An OC grown on (**i–k**) glass shows PDs and end an even low-intensity dynamin signal throughout the cell. We selected the representative images from 10 dynamin stained SZs and N > 20 APs and PDs. The scale bars are: 5 μm in (**d**), 20 μm in other images.

On the bone particle coating there were typically clusters of RPs and the pits were accompanied with SZs, APs, or SARs each with characteristic pattern of f-actin and protein localisation and membranous compartments (Figs 2, 3, 4, 5, 6 and Supplementary Figs S2, S3 and S5). More detailed studies of RPs on bone particle coating and bone slices revealed an uneven resorption pattern with smaller deep protrusions inside a larger RP (Figs 2d,e and 3d, Supplementary Fig. S2E,G,H, arrow in S2M and Supplementary videos M1–M4), all suggesting a sequence of rest and rearrangement periods between resorption bursts. However, once an OC is induced to resorb bone it continues with the task, although site of resorption may change and the cell may migrate^3^. Resorption was confirmed by measuring concentration of the c-terminal degradation fragments of type I collagen (CTX-values) (Supplementary Fig. S4). The higher CTX-value on bone slices is most likely because of more bone being available for resorption.

**Figure 7:**
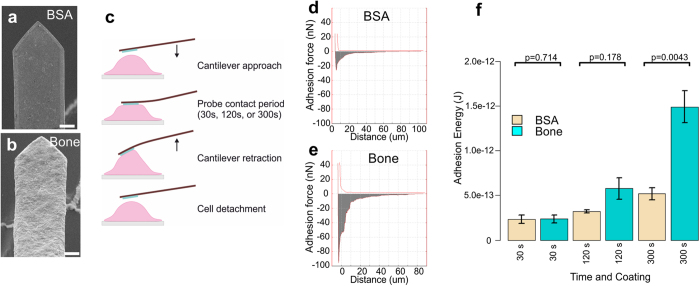
OC adhesion energy measurements with bone coated cantilevers in an AFM force spectroscopy setup. Electron micrographs of BSA coated (**a**) and human bone particle coated (**b**) tipless AFM-cantilevers. (**c**) shows a schematic diagram of an adhesion measurement that illustrates the steps of a force distance cycle. Adhesion force curves of peripheral blood-derived OCs on glass with BSA coated (**d**) and human bone particle coated (**e**) cantilevers. The shaded areas in the curves represent adhesion energies and the lowest point of the curve represents the maximum adhesion. (**f**) Comparison and statistical analysis of adhesion energy between BSA and bone coating at 3 time points, 30 s, 120 s, 300 s. Values are means ± SE (n = 5 with BSA and n = 6 with bone coated cantilevers, assessed by Mann-Whitney test). Scale bar 10 μm in (**a**,**b**).

At AP-structures we did not observe stabile filamentous actin bundles that would instinctively be taken to facilitate protracted transport of proteins and vesicles to resorption site and away into the cytoplasm, and further to the secretory domain^36^. On the bone coating, V-ATPase staining was found to localise at the plasma membrane on the site of AP, in a number of large vacuoles with strong staining for V-ATPase and small puncta of acidified lysosomes and endosomal structures (Fig. 5)^31^. However, the vacuoles were also seen above PD- and SZ-structures (Fig. 5). This suggests that SZ-structure does not necessarily depolymerise during the accumulation of acidified vesicles and possibly serves as a template to generate the RB needed for the next round of resorption. The vacuoles were surrounded by an actin capsule, again suggesting that they interact with actin network and may be transported by polymerising actin to the site of resorption. Inside RPs, the AP actin structures co-localised with intense dynamin (Fig. 6) and Arp 2/3 staining (Supplementary Fig. S5), suggesting sequestration of dynamin to these sites, intense actin dynamics and active exocytosis^37^,^38^ and endocytosis^23^,^39^. Taken together, these observations in human OCs indicate a dynamic process of resorption cycles where bursts of resorption are followed by accumulation of acidic compartments to be utilised in the next cycle of resorption, and forming and dissociation of actin structures related to the process (Figs 6 and 7, Supplementary Fig. S5 and Supplementary videos M1–M6).

### Adhesion energy measurements

As another application of the *in vitro* model, OC adhesion energies were measured in FS experiments using tipless AFM cantilevers coated with bone particles. SEM images of BSA (Fig. 7a) and bone particle coated cantilevers (Fig. 7b) show that the whole cantilever is coated with bone. FS experiments with the functionalised cantilevers were conducted in the CellHesion system (JPK) (Fig. 7c). First, spring constants of the functionalised cantilevers were measured and then OC adhesion maximum forces (highest adhesion force) and energies (area under the curve) were measured. Adhesion was stronger towards bone surface than to the control BSA coating. The representative adhesion FDCs at a 300 s time point are shown in Fig. 7d,e. The maximum adhesion force with bone-coated cantilever was around 100 nN, and around 30 nN with BSA coating. The difference in adhesion energy was even greater between the coatings in favour of bone-coated surface (Fig. 7f). The difference between adhesion energies was found to be significant (P-value 0.0043, N = 5 for BSA coating and N = 6 for bone coating) at 300 s time point. The results show that the binding to bone surface was strong and specific, and that the difference to BSA-coating became larger over the contact time suggesting specific biological activity, i.e. cell-ECM – signalling (Fig. 7f).

## Discussion

We have developed a bone particle coating method, which mimics injured human bone and serves as an *in vitro* culture substrate and an AFM probe coating material that exhibits natural biological and physicochemical microenvironment for OCs. The bone particle coating was a uniform, microscopically thin and smooth layer of bone particles that could be deposited on any surface. This layer was considerably thinner than a bone slice, and facilitated super-resolution light microscopy on resorbing OCs and easy detection of RPs. The coating was shown to be a biologically relevant mixture of inorganic and organic bone matrix including, at least, collagen fibrils. Hence, even after elaborate processing and coating, RPs, high CTX-value and innate structures of original bone e.g. filamentous collagen with D-bands were observed. Thus, the coating closely mimics natural environment for bone resorption^3^. The elasticity of the coating allowed us to coat AFM probes and expand FS experiments on OCs beyond probing interaction of single molecules^40^,^41^. We were able to compare adhesion energies in a simple, straightforward and precise manner (Fig. 7). On the bone coating we were able to utilise STED microscopy to study the actin cytoskeleton (Fig. 3 and Supplementary Fig. S1) and localise immunofluorescence signals in respect to bone with high precision in bone resorbing OCs by confocal microscopy (Figs 5 and 6 and Supplementary Fig. S5). This was made possible by the decrease of bone autofluorescence, ease of sample preparation, better optical access to the RPs and horizontal orientation of the cells and the resorption machinery in the coated samples. The even surface of the bone coating facilitated label-free identification of RP and makes calculation of the number of RPs and their volume straightforward. We observed shallow RPs indicating early stages of resorption and observed RP clusters supporting a theory of resorption being a dynamic process. Furthermore, it was confirmed that the cells were indeed OCs^15^ and could resorb bone and degrade of the organic bone matrix by CTX analysis. The CTX content of the media of both bone marrow derived OCs (Lonza) as well as peripheral blood-derived osteoclasts cultured on bone coating and bone slices were measured (Supplementary Fig. S4).

We and others^4^,^12^,^42^,^43^ have found distinct differences in OCs grown on glass and on bone-derived substrates. While OC activity has been shown on a number of substrates^8^, there is a striking difference in morphology between OCs grown on glass and bone substrates as well as in the size of the OCs and amount of nuclei per OC. On glass the OCs had very distinct morphology with a roundish, cup-like appearance with an average of 9.7 nuclei located in the middle of the cell. On bone substrates, the OCs had more irregular shape, the size of the OCs was smaller, they had only an average of 1.8 nuclei, and some resorbing cells had only one nucleus (Fig. 4). The bone coating may be an easier substrate to resorb and thus facilitate resoption by mononuclear cells. Although, observation of small RPs is easy on the bone coating, it would be more difficult on a bone slice. This significant difference and previous observations with osteoclast specific TRAcP staining^4^,^44^ on mononuclear cells indicate that fusion of OC precursors would not be an essential step in formation of resorbing mature human OCs. It would appear that without stimulus to commence resorption, osteoclasts on artificial substrates tend to fuse to form larger cells with more nuclei (Fig. 4a). While majority of observations in literature match the classical OC definition (TRAcP positive and 3 or more nuclei), it would appear that on bone coating there are bone resorbing cells that do not have more than three nuclei and there are multinuclear TRAcP positive foreign body giant cells that are not able to resorb bone^15^. In the resorbing OCs that had several nuclei, typically one, slightly larger nucleus was found very close to the site of resorption (Fig. 4a,b). Conversely on glass the nuclei were located further away from the cell-glass interface, implying a lower level of activity.

On bone we were able to visualise cells exhibiting a dynamic mixture of actin structures i.e. PD, SAR, SZ and AP (Fig. 2 and Supplementary Fig. S2), matching previous findings^3^,^19^,^32^,^45^. The PD-structures were clearly visible on glass and vitronectin-coated glass, and also on bone, although their abundance was reduced. This finding may indicate that upon coming to contact with the physicochemical substrate suitable for resorption the mechanosensing function of PDs is fulfilled^46^,^47^ or that on bone OC prefers different adhesion mechanism as a result of biological recognition^26^. The SZ was apparent only in some of the cells cultured on bone, both bone coating and slices, while APs and SARs were more common. We have visualised V-ATPase (Fig. 5)^48^, Arp2/3 (Supplementary Fig. S5)^25^,^49^, dynamin (Fig. 6)^23^,^50^ and membranous structures^15^ at RP at AP-structures to indicate active on-going resorption (Supplementary Figs S3–S4). In addition to this, we observed a medium intensity of V-ATPase in the middle of a SZ (Fig. 5). Further away from the bone interface, we observed vacuoles filled with V-ATPase. In OCs grown on glass, we observed only staining of vacuoles and lysosomes and endosomal structures, but no accumulation at the glass interface. Our data supports past studies showing that V-ATPase generates the low pH needed to dissolve bone^31^,^48^, but also suggest that pre-acidified vacuoles could be transported to APs at resorption sites during active resorption.

Dynamin staining was spread throughout the plasma membrane, however, an intense staining at AP-structures was observed (Fig. 6). Previously it has been shown that dynamin blocker dynasore inhibits resorption^50^, and that RBs are the bone resorbing organelles^14^,^15^; we observed the strongest association between actin and dynamin at the AP-sites, and RPs aligned consistently with APs and SARs, therefore we presume APs signify RBs. Moreover, an overlay image of dynamin and f-actin shows dynamin localising at the puncta in actin network (Fig. 6). This data suggest that there is active vesicular transport at the AP and less at the other actin structures. The Arp2/3 localisation in filaments at APs (Supplementary Fig. S5) may indicate that branched actin could be involved in generating the forces to form protrusions and drive endocytic and exocytic vesicle transport to deliver acidic vacuoles to RB and to remove resorption products from RB.

At the RB, prior EM data has shown multiple deep invaginations that have been associated with endocytosis of resorbed material and exocytosis of ions and proteases^45^,^30^. Staining the OC membranes we observed membrane protrusions aligning with f-actin at AP along with other membranous structures, again indicating that the AP indeed is the RB, and on SZ we found only plasma membrane and a wide band of membrane free cytoplasm (Supplementary Fig. S3), an observation consistent with previous reports^15^. In most cases there was no SZ associated with observed RPs or APs, however, with lager RPs, a SZ was typically observed (Fig. 2). The lack of SZ associated with APs and RPs (Figs 2 and 5 and Supplementary Fig. S2) indicates unconnected formation or fast and possibly independent dynamics of SZ and RB structures^3^. Moreover, an OC grown on bone coating and exhibiting a SZ does not consistently show accumulation of dynamin at the SZ or at its centre (Fig. 6). Hence, SZ and RB appear to be separate structures with different biological roles and at least existence of SZ does not necessitate existence of RB^15^. Further experiments are needed to establish the mechanisms through which the membrane dynamics are regulated at resorption sites and how does the transport relate to actin micro-domains.

We measured adhesion energy of OCs to bone and BSA coated probes at three different time points: 30 s that permits interaction of receptors already expressed on OC surface, 120 s that permits adhesion-related signalling to progress further and vesicular transport to take place and 300 s that allows signalling that is likely to reach nuclear receptors, and over the course of the experiment, 15 FDCs (75 min), for transcription to take place^51^. The OCs had higher average adhesion energy when adhering to bone coating than to BSA coating with each contact time. The most significant difference was measured when the cells were allowed to adhere to the probe for 300 s (Fig. 7). According to expectations, we measured also an increase of adhesion to BSA coating as the contact time increased, but the slope of this increase was less than the increase of adhesion observed to bone coating. This adaptive adhesion suggests a process of bone recognition and signalling, followed by transcription, translation and translocation of the proteins, all specific to OC-bone contact^52^,^53^.

**Figure 8:**
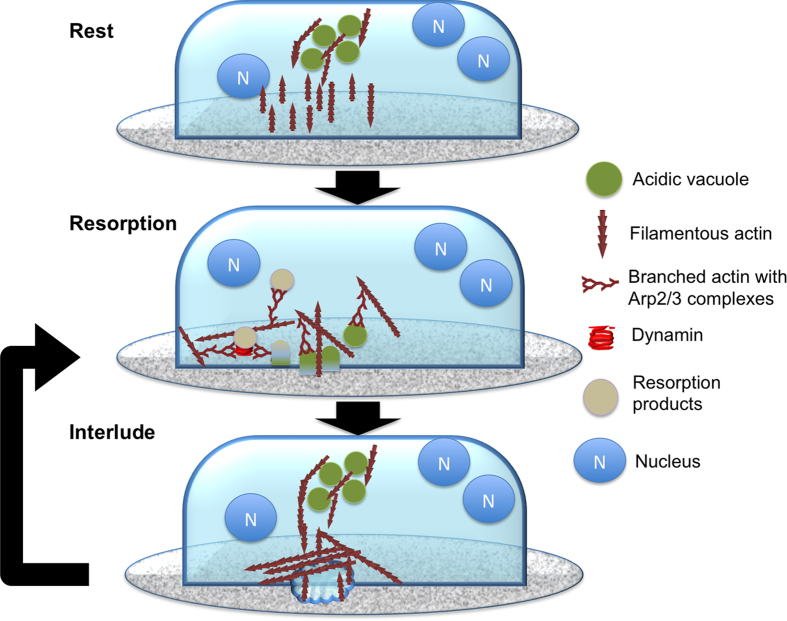
Schematic illustration of the proposed mechanism of resorption cycle and vesicular transport during the cycle. Actin encapsulated vacuoles filled with V-ATPase and proteases are transported to plasma membrane forming the RB area. Filamentous and dynamic branched actin forms the observed AP structures at RB during this process. The vacuoles are likely to be acidified and when fusing with plasma membrane they initiate a rapid decrease of pH in the microenvironment. The V-ATPases remain on the membrane and may maintain the acidic microenvironment for a while. During this process begins endocytosis and subsequent removal of the resorption products. This process needs both dynamin and Arp2/3, and hence, the forces driving the process seem to be generated by branched actin polymerisation. After the active phase stops V-ATPase and both low level of actin remain on the plasma membrane and the resorption may be re-initiated at the same location by fusion of more V-ATPase filled acidified vesicles. The mechanism we propose describes resorption as a dynamic process that relies bursts of acidified vesicles and explains the emergence of clusters of small RPs.

We have shown that actin structure above RPs did not have stabile vertical actin bundles but a local network of lateral fibrils. These fibrils could mediate binding to the calcified substrate to be resorbed, create protrusions and exert the forces required to drive vesicular transport (Fig. 3)^54^,^55^,^56^. The presence of bright dynamin signal at the nodes of the actin network and string-like Arp2/3 staining suggest that these forces are likely to be exerted by branched actin polymerisation (Fig. 6 and Supplementary Fig. S5)^33^,^38^,^57^. This conclusion is further corroborated by visualisation of high abundance and string-like morphology of Arp2/3 complexes at RB, whereas in PDs and SZs the filament length of Arp2/3 was shorter (Supplementary Fig. S5). The presence of Arp2/3 staining has been linked with dynamic actin polymerisation and vesicular transport, and in the case of OCs it could serve a role in both exocytosis of acidified vacuoles and endocytosis of resorbed material. We propose that resorption is carried out in temporally controlled cycles where acidified vacuoles are accumulated within the OC, vacuoles are delivered to RB to generate acidic microenvironment, and resorption products are removed from the RP by endocytosis (Fig. 8). Forces to drive this cycle are generated by dynamic actin polymerisation and possibly by myosin 10 mediated transport along actin^26^.

The species-specific bone coating facilitates super-resolution LM throughout the coating with STED microscope and adhesion measurements with AFM on live osteoclasts, all in a relevant biological context. The higher detail of LM was achieved due to the microscale thickness of the novel coating that reduced scattering and autofluorescence. The more detailed imaging at bone interface facilitated insights to the resorption process and supports dynamic actin reorganisation and that RB visualised, as AP is the necessary organelle to resorb bone. The application of the coating for FS is made possible by its elasticity and ability to form a dense coverage, even on small surfaces. Our study puts forth a temporally controlled mechanism where resorption progresses in a series of cycles maintaining the acidic microenvironment for relatively brief periods at a time through release of acidified vacuoles (Fig. 8).

## Methods

### Osteoclast cultures

Two types of OC cultures were utilised: OC cultures were established from human peripheral blood, and from human bone marrow-derived CD34+ stem cells (Lonza, Walkersville, USA). Blood-derived human OC cultures (N = 33) from peripheral blood of healthy volunteers (N = 3) were performed as previously described^34^,^35^. The study was approved by Ethics Committee of Southwest Finland Hospital District, and the participants gave their informed written consents to osteoclast cultures. The study was carried out in accordance with the guidelines set for research on human tissue by Turku University Hospital and University of Turku. First monocyte fraction was isolated with Ficoll gradient centrifugation according to the manufacturer’s protocol. Then the monocytes were seeded to coverslips and differentiated to multinuclear OCs in the presence of medium containing alphaMEM (GIBGO, Grand Island, NY, 41061-029), 10% iFBS (GIBCO, Grand Island, NY, 16000-044), 10 mM HEPES (Sigma-Aldrich, H0887), 1:100 penicillin-streptomycin, RANKL (20 ng/ml, Peprotech, Rocky Hill, NJ, 310-01) and M-CSF (10 ng/ml, R&D systems, Minneapolis, MN) for 8–10 days. Half of the medium was changed to fresh one after 3–4 days in order to preserve auto- and paracrine signalling molecules.

Human bone marrow-derived CD34+ stem cells were cultured according to provider’s protocol^58^. The CD34+ stem cells were suspended in culture medium and allowed to attach to glass, bone slice, or functionalised glass surfaces. The culture medium (containing 10% FBS, OCP BulletKit^®^ Lonza,) was supplemented with 33 ng/ml M-CSF and 66 ng/ml RANK-ligand (Lonza). After the osteoclast differentiation period on day 7, all culture medium was removed and fresh medium was added into the wells. The mature osteoclasts were cultured for an additional 3 days, during which they were allowed to resorb bone. This medium was then analysed for C-terminal cross-linking telopeptides of type I collagen (CTX) (Supplementary Methods). All cells were on the respective substrata throughout the whole culture period.

### Bone powder for cell culture substratum

Use of expired, banked human bone was approved by Northern Ostrobothnia Hospital District Ethical committee (statement 16/2009) and Valvira, National Supervisory Authority for Welfare and Health (Dnro 220/05.01.00.06/2009). The procedure to extract the tissue was conducted in accordance with guidelines approved by Northern Ostrobothnia Hospital District Ethical committee. Donors had given their informed written consent to use the material in academic research. Cortical human bone from three different donors was mechanically pre-ground to produce millimetre scale powder. The pre-processed bone was further milled in ethanol suspension with a planetary ball mill (Fritsch Pulverisette 6, Idar-Oberstein, Germany) to reach a particle size of 10–1000 nm, in order to produce a smooth and thin coating. Sedimentation in ethanol was used to isolate a 10–200 nm-sized fraction of particles for coating. The milling balls used were 2 mm diameter ZrO_2_ particles from Retch (Haan, Germany). After milling the bone particle suspension was allowed settle for 15 min and the soluble fraction was removed. This fraction was further allowed to sediment for 2 h. After second sedimentation step the soluble fraction was taken out and allowed to sediment for 16 h. The third fraction of particles remaining in suspension was then removed and used for coating the selected surfaces.

### Coating of coverslips

Cleaned coverslips were coated with poly-l-lysine by incubating in 0.01% poly-l-lysine solution (Sigma-Aldrich Corporation, St. Louis, USA, P1524) overnight. Then, coverslips were coated with bone particles in buffer with 10 mM PO_4_, 0.025% (w/v) Triton X-100, and 0.05% (w/v) BSA. Prior to coating the bone particles were removed from ethanol we used as the milling suspension and re-suspended to coating solutions. We tested different coating solution compositions, e.g. 1) 10 mM PO_4_, 0.05% BSA and 0.025% Triton X-100, 2) ethanol, 3) ion exchanged water, 4) 10 mM PO_4_ and 0.1% milk, 5) 10 mM PO_4_, 0.1% milk and 0.1% Triton X-100, and 6) 10 mM PO_4_ and 150 mM NaCl. After the coating suspension had dried the coverslips were carefully washed in water for 30 minutes and then dried completely. Finally the bone coating was fixed with 4% paraformaldehyde (PFA) for 20 minutes and stored at 4 °C. The vitronectin coating was prepared using the same protocol with a solution of 1 μg/ml vitronectin in water. All incubations were carried out at room temperature.

### Coating of AFM cantilevers

For adhesion energy measurement, tipless AFM cantilevers (CSC12/tipless/no Al, MikroMasch®, Innovative Solutions Bulgaria Ltd., Sofia, Bulgaria) were coated with bone particles. Prior to the bone particle coating, cantilevers were treated with APTES ((3-aminopropyl)triethoxysilane)/ethanolamine vapour^59^. The APTES coated cantilevers were subsequently coated with bone particles in the aforementioned bone solution. The cantilevers were incubated in the bone solution overnight, fixed with 4% PFA for 20 minutes, washed gently in PBS for 30 minutes, and stored in PBS at 4 °C.

### Cell adhesion measurement

OC adhesion force to coated AFM cantilever was measured with JPK AFM equipped with a CellHesion module (JPK Instruments) on a Zeiss LSM510 microscope (Carl Zeiss Microscopy). During the measurements, cells were kept at 37 °C with a BioCell module (JPK). Prior to the force measurements, AFM cantilevers were calibrated with measured deflection sensitivity and spring constant by the instrument calibration program. For the force measurements, OCs with 4–6 nuclei were selected with fluorescence microscopy by staining the nuclei with Hoechst 33342. Excitation was with a mercury lamp and fluorescence was detected with CCD camera. After a functionally coated cantilever was placed on the selected cell, instrument was run at force spectroscopy mode with relative deflection of 2 V and z-range of 70–100 um, depending on vertical dimension of the OCs. Adhesion force curves were recorded at three different extraction delay time, 30, 120, and 300 seconds to give different time periods for cellular responses. The measurements with each contact time were repeated at least 10 times on each cell with a single cantilever and then a mean adhesion was calculated. The data represents averaged adhesion energies of OC derived from the blood of 5 healthy volunteers. The experiment was conducted with bone particle coated and BSA coated cantilevers. Measured force curves were analysed to calculate adhesion energy by JPK Data Processing software (JPK Instruments), and the statistical analysis was performed with Mann-Whitney test available in R (version 3.0.3, GNU Software).

### Confocal and STED fluorescence microscopy and labelling of the OCs

More details on labelling and imaging are provided in supplementary methods and here we briefly summarise the experimental procedures. Cells were labelled with 2.5 μg/ml anti-dynamin mouse antibody, 10 μg/ml anti-arp2 rabbit antibody or 2 μg/ml anti-V-ATPase rabbit polyclonal antibody recognising all isoforms for 1 h in PBS containing 0.5% BSA. The secondary labeling was conducted for 1 h with anti-mouse or anti-rabbit alexa488 in PBS containing 0.5% BSA, along with 200 ng/ml phalloidin-Star635 and 10 ng/ml PicoGreen. To stain membranes we used 5 μg/ml of lipophilic DiI. The cells were then washed and embedded in mowiol. All images were acquired with a Leica SP5 STED microscope, equipped with pulsed Ti: Sapphire depletion laser MaiTai HP and 635 nm pulsed laser and an avalanche photo diode (APD) detector at 665–705 nm range. STED microscopy images were processed using DeconvolutionLab plugin^60^ (Richardson-Lucy with TV regularisation algorithm, regularisation parameter 0.005 and 20 iterations, for panel 3O only 4 iterations were used) for ImageJ with experimentally measured PSF.

## Additional Information

**How to cite this article**: Deguchi, T. *et al.* *In vitro* model of bone to facilitate measurement of adhesion forces and super-resolution imaging of osteoclasts. *Sci. Rep.* **6**, 22585; doi: 10.1038/srep22585 (2016).

## Supplementary Material

Supplementary Movie 1

Supplementary Movie 2

Supplementary Movie 3

Supplementary Movie 4

Supplementary Movie 5

Supplementary Movie 6

Supplementary Movie 7

Supplementary Information
