# Supplementary material for: In vitro model of bone to facilitate measurement of adhesion forces and super-resolution imaging of osteoclasts
Source: Sci Rep. 2016 Mar 3;6:22585. doi: 10.1038/srep22585 (PMC4776281; doi:10.1038/srep22585)
Supplement: Supplementary Information [file srep22585-s8.pdf]

## *In vitro* model of bone to facilitate measurement of adhesion forces and super-resolution imaging of osteoclasts

Takahiro Deguchi, Maria H. Alanne, Elnaz Fazeli, Katja M. Fagerlund, Paula Pennanen, Petri Lehenkari, Pekka E. Hänninen, Juha Peltonen and Tuomas Näreoja

### Online supplemental material

Supplementary fig. S1 shows comparison of resolution increase in STED imaging of OCs cultured on bone coating and bone slice and stained with phalloidin-Star635.Supplementary fig. S2 shows the various actin structures present in OCs grown on bone-derived culture substrata. Supplementary fig. S3 shows lipophilic DiI staining of membranous compartments associated with AP and SZ structures in OCs cultured on bone coating. Supplementary fig. S4 shows a CTX measurement to quantify resorption done from culture mediums used to culture OCs on bone coating, bone slice and glass and from wells where bone coating was soaked in medium for equal time. Supplementary fig. S5 shows Arp2/3 complex localization in relation to f-actin in human OCs exhibiting APs, SZs and PDs. The manuscript is supplemented with Movies 1-7 to better illustrate the axial dimensions of figures 2, 4-7 and Supplementary fig. S1, S3 and S5. Movie 1 (from Fig. 2) shows typical morphology of OCs cultured on coverslip, vitronectin coated coverslip, bone coated coverslip and on bone slice. Resorption can be observed on bone and podosomes on glass and virtronectin surfaces. Movie 2 (from Supplementary fig. S2) shows resorption structures SZ, SAR, and AP on bone coating and on bone slice. Movie 3 (from Fig. 4) shows that the substrate material affects both localization and organization of nuclei. When resorption is observed one of the nuclei is located close to the site of resorption and on coverslip nuclei are clustered together deeper inside the cell. Movie 4 (from Fig. 5) V-ATPase is accumulated at APs, RPs, in the middle of SZs, when APs are there, but not at SZs. When APs are not observed the V-ATPase can be seen accumulated in vacuoles. Movie 5 (from Fig. 6) Strong dynamin staining is observed at RB while to lesser degree it is present throughout the cell. Movie 6 (from Supplementary fig. S5) Strong Arp2/3 staining is observed at all resorption related actin structures and on PD indicating active actin nucleation and thereby force generation. Movie 7 (from Supplementary Fig. S3) showing membranous compartments present at AP and SZ.

### Supplemental experimental procedures

#### Fluorescence labelling of OCs

Cells were first fixed with 4% PFA in PBS for 20 min, permeabilised in 0.2% Triton X-100 in PBS for 20 min, and blocked in 5% BSA in PBS for 45 min. Cells were labelled with 2.5 µg/ml anti-dynamin mouse antibody (BD Biosciences, Clone 41/Dynamin catalogue No. 610245), 10 µg/ml anti-arp2 rabbit antibody (abcam, ab47654), or 2 µg/ml polyclonal anti-V-ATPase raised against v0 a1 isoform that cross-reacts with other isoforms. Sequence similarity to a4 is 73% and to a3 53% (Santa Cruz Biotechnology, sc28801) for 1 h in PBS containing 0.5% BSA. The secondary antibody staining was for 1 h with anti-mouse or anti-rabbit alexa488 (Sigma-Aldrich) in PBS containing 0.5% BSA, 200 ng/ml phalloidin-Star635 (Abberior) and 10 ng/ml PicoGreen (Life Technologies Europe BV) To stain membranes we used 5 µg/ml of lipophilic DiI. Three washes were performed at the end of each step and all incubations were carried out at room temperature. The cells were then embedded

with mowiol on slides. For AFM cell adhesion force measurements, cells were labelled only with DNA fluorophore 1 ng/ml Hoechst33342 (Lifetime technologies) in cell culture medium at 37°C for 20 min. Live cells were stored at 37°C on coverslips in clear Hepes-buffered alphaMEM until measurements. In order to image membrane, cells were labelled with 5 µg/ml of lipophilic DiI (Thermo Fisher Scientific Inc., USA) along with phalloidin-Star635. The same staining protocol with other immunostaining protocols was used with the exception that Triton X-100 was replaced with 0.5 % saponin and used throughout the permeabilisation, staining and washing solutions to preserve lipids in membrane, and the sample was embedded in water instead of mowiol.

### Confocal and STED fluorescence microscopy

All images were acquired with a Leica SP5 STED microscope, equipped with pulsed Ti: Sapphire depletion laser MaiTai HP (Leica Microsystems GmbH, Mannheim, Germany and Spectra-Physics, US). Atto647N and Star635 were excited by 635 nm pulsed laser (LDH-P-C-640B, PicoQuant, Berlin, Germany) and fluorescence was collected with an avalanche photo diode (APD) detector at 665-705 nm range. PicoGreen was excited at 488 nm and detected in 500-560 nm by photomultiplier tubes. For imaging bone surface, reflection light from 456 nm laser was collected at 450 – 470 nm as well as transmission light was collected by photomultiplier tubes. For super-resolution STED imaging, Star635 was excited by 635 nm pulsed laser and depleted at 770nm. Images were acquired with an oil immersion objective (N.A.1.4 100x Oil, Leica) and the confocal pinhole was set to one airy unit, with a line-scan speed of 600 Hz, a line averaging of either 8 or 16. The pixel-size (sampling) was set to satisfy the Nyquist sampling requirement. STED resolution was checked through bone coating (Supplementary fig. S2), showing full width at half maximum of approx. 60nm and resolving two peaks approx. 120 nm apart, demonstrating resolution improvement from confocal microscopy. The images were processed using ImageJ 1.49s. Confocal fluorescence images were smoothed (with Gaussian Blur, sigma 1.00 or median filter, 2 pixel radius) and intensity was adjusted for better visualization. STED microscopy images were processed using DeconvolutionLab plugin[37] (Rechardson_Lucy with TV regularization algorithm, Regularization parameter 0.005 and iteration 20, for panel 3O only 4 iterations were used) for ImageJ with experimentally measured PSF.

### Bone surface imaging by Scanning Electron Microscopy and AFM imaging

For Scanning Electron Microscopy (SEM) characterization of bone coating, the sample surface was coated with thin layer of carbon by vaporization and the scans were performed with Leo 1530 Gemini (Zeiss) equipped with an UltraDry Silicon Drift Detector (Thermo Scientific). The magnification of the image corresponds to a Polaroid 545 print with the image size of 8.9x11.4 cm.
For Atomic Force Microscopy (AFM) on bone surface, images were acquired with Agilent 5500 SPM (Agilent Technologies) in AC AFM mode with NSC19 probe (MikroMasch®, Innovative Solutions Bulgaria) at 2048 pixels x 2048 pixels with a driving frequency of 14 kHz and scanning speed of 24 um/s.

### C-terminal cross-linking telopeptides of type I collagen (CTX) detection analysis

CTX was measured in the culture media collected at day 8-10 using the Crosslaps for Culture ELISA (IDS Ltd, Boldon, UK) and VICTOR2™ Multilabel Counter (PerkinElmer,

Waltham, MA, USA). Thickness of the coating is around one per cent of the thickness of a traditional sawed bone slice. This provides greatly improved optical transmission that allows imaging through the coating also in a 96-well format. In control OC samples on glass, bone slice without OCs or bone particle coating without OCs (Supplementary fig. S4), low levels of CTX from background were observed. The similar CTX values from glass with OCs and bone particle coating without OCs excluded the possibility that the CTX levels would have been affected by the milling protocol. The bone particle coating can be deposited onto any relevant surface material with slight surface chemistry variations, and therefore it constitutes a widely applicable 3D-culture model for bone biology research.

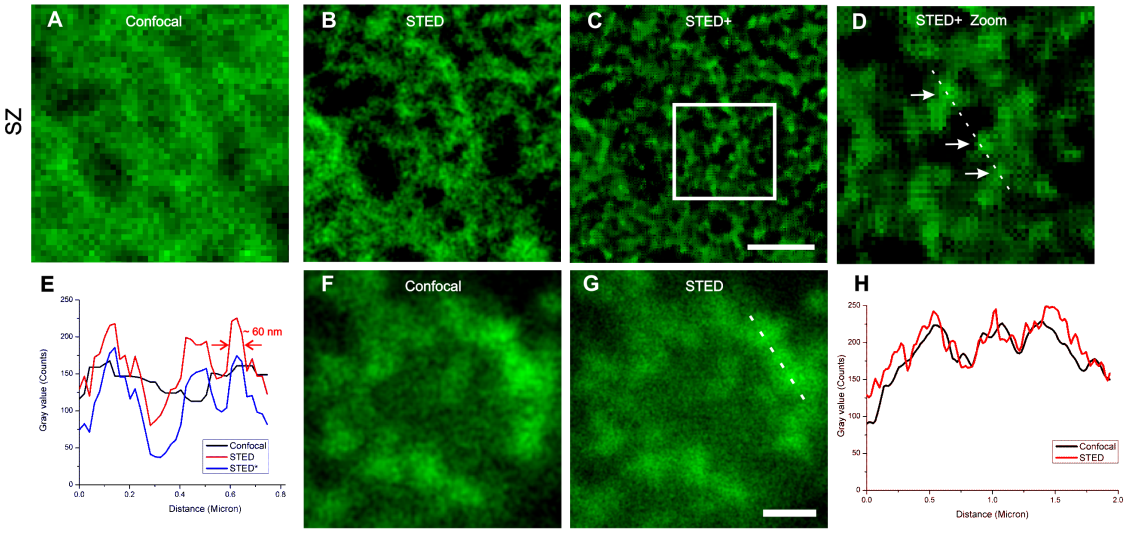


**Supplementary figure S1. Super-resolution STED imaging on bone coated coverslips at bone interface exhibiting a SZ structure. Comparison of confocal (A), STED (B) and deconvolved STED (C) images are presented and a measurement of single fibre full-width at half-maximum in the graph (E) demonstrated the resolution improvement. Site of the measured cross-section is shown in zoomed inset (D). Visualization of detailed structures in the network of f-actin is possible in super-resolution data on the new *in vitro* model. Scale bar 1 µm.**

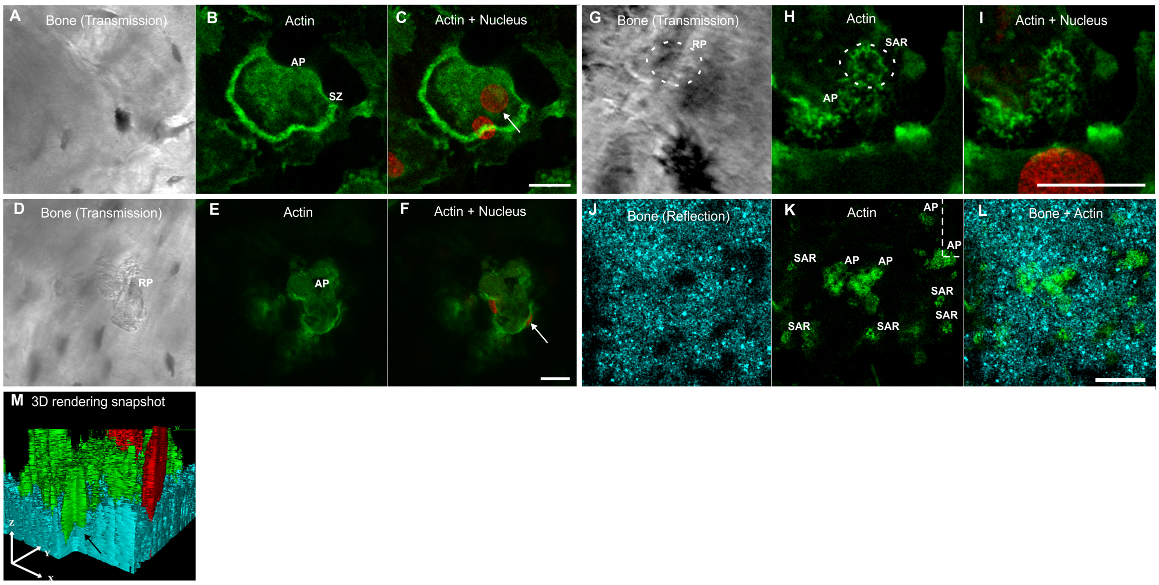


**Supplementary figure S2. Actin (green) morphology of resorbing peripheral blood-derived OCs on human bone reveals various structures coinciding with RPs. Filamentous actin formed elaborate structures both bordering the RPs and in the resorption pits. In the classical case (A-C) a SZ surrounds an AP colocalizing with a RB-structure.**

However, all RPs were not associated with SZ, but AP structures were still present in these cases (B-L). Typically AP structures without SZ were smaller with wide variation in sizes. In many cases these APs (B-L) were protruding into bone substrate. In panel (M) is a 3D rendering from dotted line in (K) showing actin (green) at arrow penetrating into bone (cyan), with nearby nuclei (red). Interestingly, with the blood-derived OCs we were able to visualize fewer sealing zones on bone coating. The lower abundance of sealing zones may be due to more dynamic nature of resorption on the thin and particulate coating that would favour short resorption bursts. The optimized OCs showed the aforementioned small rings and APs, and also SZs, on both bone substrates. Thus, the higher activity of the optimized OCs could sustain protracted resorption irrespective of the coating type. This adds to the evidence that OCs may be able to resorb bone without forming a SZ and that resorption is a dynamic process which adapts to the substrate being resorbed[(3)(32)]. The z-scale in (M) is ten times enhanced. For APs and SARs with both blood- and bone marrow derived OCs grown on both substrates, the representative features were selected from N>20 examples. Scale bar is 20μm.

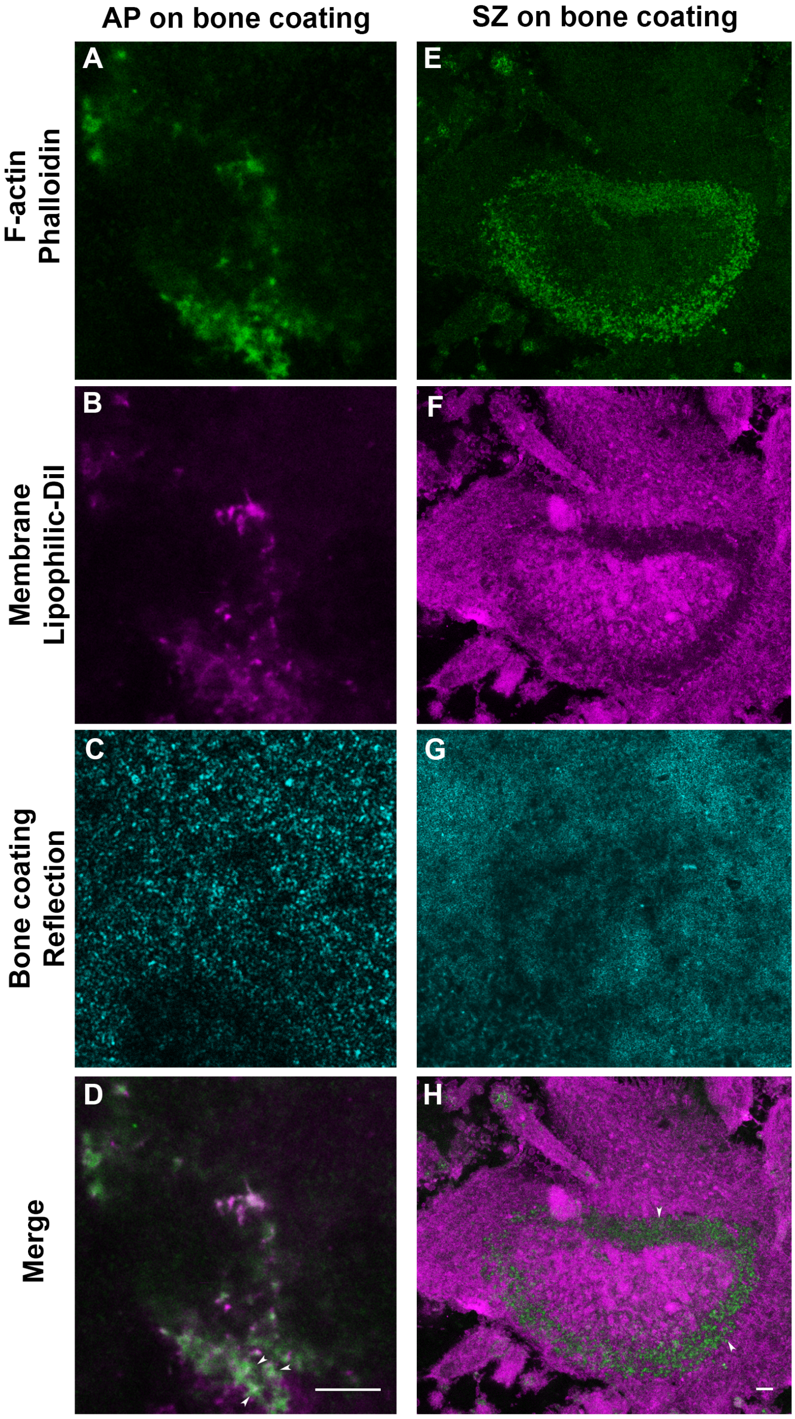

Supplementary figure S3. Membranous compartments are present at AP (A-D), but absent on SZ (E-H). Lipophilic-DiI staining (magenta, B and F) of membranous compartments e.g. plasma membrane, endosomes and ER, f-actin (green, A and E) and bone (cyan, C and G) of OCs on bone coating. On AP membrane protrusions are seen to coincide with actin staining (D, arrowheads) and abundance of other membranous compartments are present including endosomal structures (see supplementary Movie 8). At SZ only a thin plasma membrane layer is observed and above SZ there is a 1 µm thick belt devoid of membranous structures (H, arrowheads), like previously shown with transmission EM[15]. Panels A-D are a single slice to illustrate protrusions at bone interface and panels E-H is a maximum projection of 1 µm to illustrate the volume devoid of membranous structures. Scale bar 5 µm.

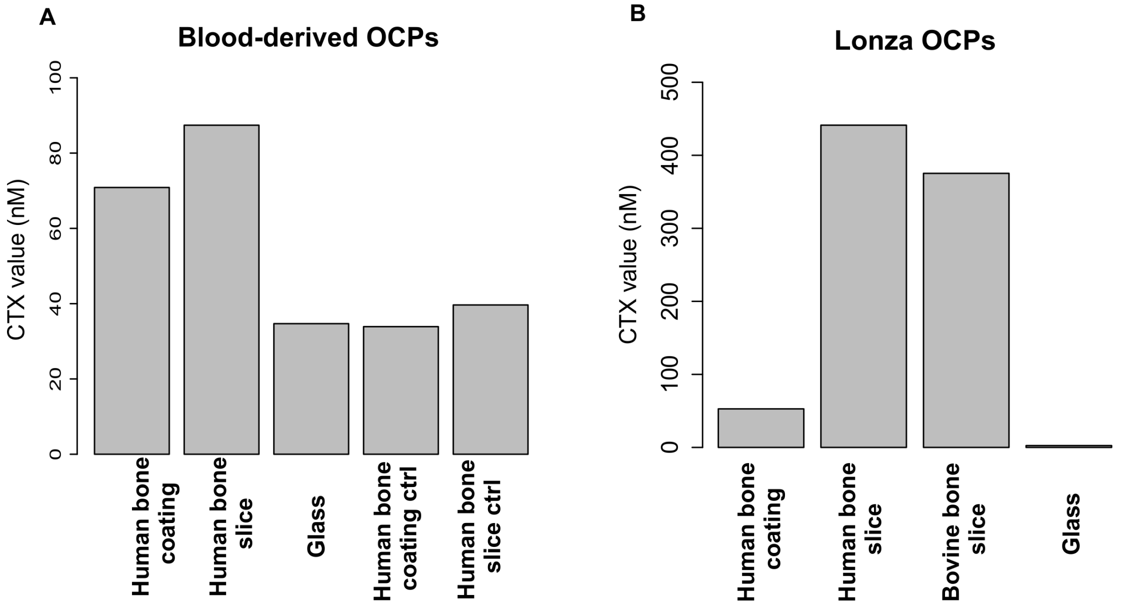


Supplementary figure S5. To confirm bone resorption by OCs on both substrates, bone particle coating and bone slice, the concentration of a bone degradation product, type I collagen C-terminal cross-linking telopeptide (CTX), was measured. Bone resorption activity on different substrates from CTX analysis. CTX values of peripheral blood-derived OCs (A), compared on human bone slice, human bone particle coating, and on glass are shown. As control experiments, CTX values from human bone slices and human bone particle coating without OCs are also shown. CTX values of bone marrow derived OCs (B) compared on human bone slice, bovine bone slice, human bone particle coating, and on glass are shown. The results are from samples pooled from 4 independent replicates for blood-derived cells and 5 replicates for commercial cells. The two OC types, blood-derived and optimized OCs, exhibited different activity profiles. Both OC types were found to be actively resorbing on bone coating and bone slices, and the higher CTX-value on bone slices is most likely because of higher amount of bone being available for resorption. The CTX levels were high with OCs on bone particle coating and corresponded to those measured from bone slice –samples, when taking into account that the total amount of bone in the bone particle coating was around one per cent of bone in a bone slice. These results confirm that there was an active on-going resorption on bone-coated samples with peripheral blood-derived OCs, even though the activity was lower.

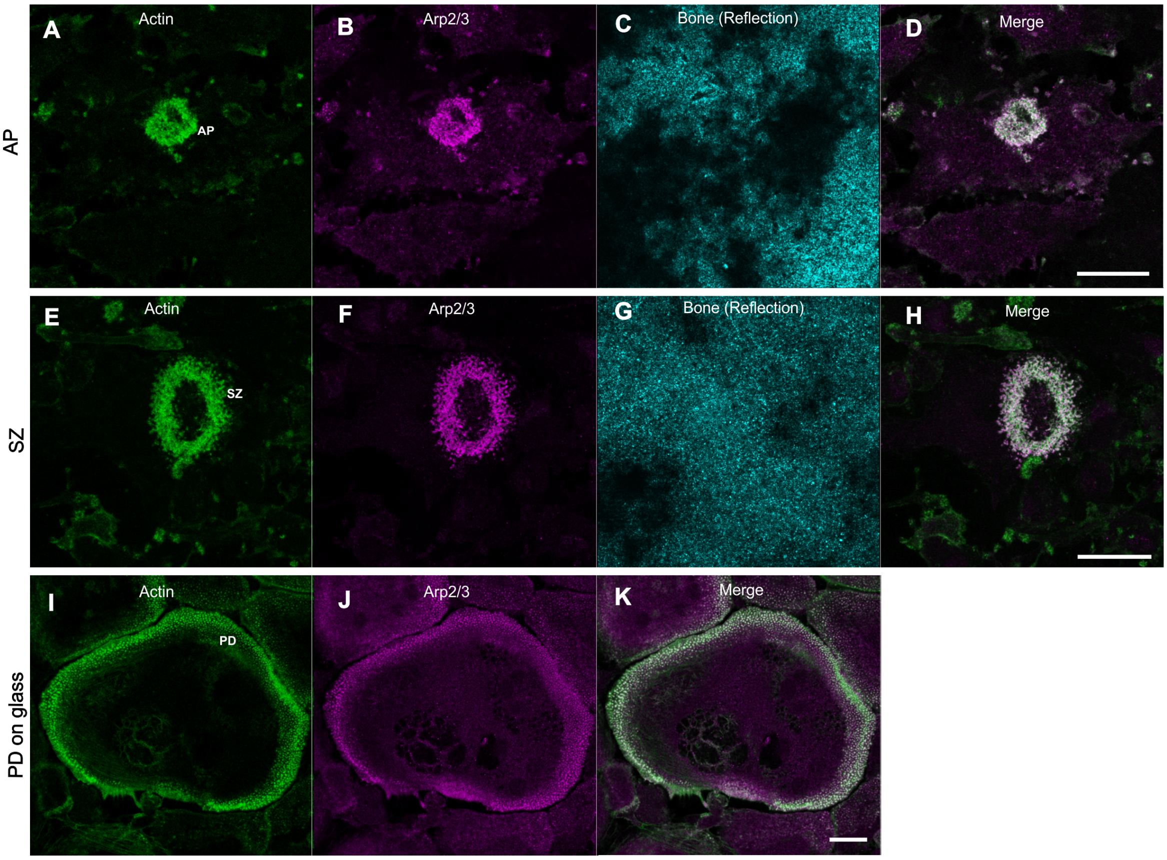


**Supplementary figure S5. Arp2/3 localization in human OCs exhibiting APs, SZs and PDs. A-D) AP structure of filamentous actin (green) that shows an intense Arp2/3 staining (magenta) and cyan showing the bone interface. The Arp2/3 signal is organized in filaments, and despite of considerable overlap, all actin signals do not fully co-localise with Arp2/3. Most of the co-localisation is seen at high intensity puncta and filaments. We selected the representative images from 7 Arp2/3 stained SZs and N>20 APs and PDs. Scale bar: 20 µm.**

**Movie 1 (from Fig. 2). Typical morphology of OCs cultured on coverslip, vitronectin coated coverslip, bone coated coverslip and on bone slice. Resorption can be observed on bone and podosomes on glass and virtronectin surfaces. F-actin was visualized phalloidin-Star635 staining (green). Bone slices surface is shown with transmission light (grayscale) and bone particle surface is shown reflected light (cyan). The z-stacks were taken from the cell bottom (glass or bone surfaces) to the cell top, section thicknesses were 250 nm, scale bars are 20 µm and playback rate is 5 frames/s.**

**Movie 2 (from Supplementary fig. S2). Resorption structures SZ, SAR, and AP on bone coating and on bone slice in more detail. All structures were visualized on RPs or in vicinity of RPs. F-actin was visualized phalloidin-Star635 staining (green). Bone slices surface is shown with transmission light (grayscale) and bone particle surface is shown reflected light (cyan). The z-stacks were taken from the cell bottom (glass or bone surfaces) to the cell top, section thicknesses were 250 nm, scale bars are 10 µm and playback rate is 5 frames/s.**

**Movie 3 (from Fig. 4). Location and organization of nuclei is affected by the substrate material. When resorption is observed one of the nuclei is located close to the site of resorption and on coverslip nuclei are clustered together deeper inside the cell. F-actin outlining the cell boarders and resorption structures was visualized phalloidin-Star635 staining (green) and the nuclei were stained Hoechst 33342. The z-stacks were taken from the cell bottom (glass or bone surfaces) to the cell top, section thicknesses were 250 nm, scale bars are 20 µm and playback rate is 5 frames/s.**

**Movie 4 (from Fig. 5). V-ATPase is accumulated at AP and RP, but not at SZ or PD. When APs are not observed the V-ATPase can be seen accumulated in vacuoles deeper inside the OC. F-actin was visualized phalloidin-Star635 staining (green) and V-ATPase with a monoclonal antibody–secondary antibody combination (magenta). The z-stacks were taken from the cell bottom (glass or bone surfaces) to the cell top, section thicknesses were 250 nm, scale bars are 20 µm and playback rate is 5 frames/s.**

**Movie 5 (from Fig. 6). Strong dynamin staining is observed at AP while to lesser degree it is present throughout the cell. Similar strong staining was not observed at SZ or PD. F-actin was visualized phalloidin-Star635 staining**

**(green) and dynamin (isotypes 1 and 2)) with a monoclonal antibody–secondary antibody combination (magenta). The z-stacks were taken from the cell bottom (glass or bone surfaces) to the cell top, section thicknesses were 250 nm, scale bars are20 µm and playback rate is 5 frames/s.**

**Movie 6 (from Supplementary fig. S5). Strong Arp2/3 staining is observed at all resorption related actin structures and on PD indicating active actin nucleation and thereby force generation. F-actin was visualized phalloidin-Star635 staining (green) and Arp2/3 with a monoclonal antibody–secondary antibody combination (magenta). The z-stacks were taken from the cell bottom (glass or bone surfaces) to the cell top, section thicknesses were 250 nm, scale bars are 20 µm and playback rate is 5 frames/s.**

**Movie 7 (from Supplementary Fig. S3). Membranous compartments are present at AP, but absent on SZ. Lipophilic-DiI staining (magenta) of membranous compartments e.g. plasma membrane, endosomes and ER, f-actin (green) and bone (cyan) of OCs on bone coating. At AP membrane protrusions are seen to coincide with actin staining and abundance of other membranous compartments are present above the AP including endosomal structures. At SZ only a thin plasma membrane layer is observed and above SZ there is a 1 µm thick belt devoid of membranous structures, like previously shown with transmission EM[(15)]. The z-stacks were taken from the cell bottom (glass or bone surfaces) to the cell top and section thicknesses were 250 nm, scale bars are 20 µm and playback rate is 5 frames/s.**
